# Cleft palate repair: velopharyngeal closure before and during the basal tone

**DOI:** 10.1590/S1808-86942010000200007

**Published:** 2015-10-19

**Authors:** Giseane Conterno, Carla Aparecida Cielo, Vanessa Santos Elias

**Affiliations:** MSc in Human Communications Disorders, Speech and hearing therapist; PhD in Applied Language - Pontifícia Universidade Católica do Rio Grande do Sul, Adjunct Professor - Department of Speech and Hearing Therapy - Universidade Federal de Santa Maria-RS; MSc in Human Communication Disorders - Universidade Federal de Santa Maria-RS, Adjunct Professor - Speech and Hearing Therapy Department - Feevala-RS

**Keywords:** soft, cleft palate, palate, voice

## Abstract

Patients with palatine fissure have inadequate velopharyngeal closure (VPC), with consequent vocal hypernasality which can be reduced by a basal tone.

**Aim:**

to compare VPC during a basal tone with the emission of a modal recording, in patients with repaired post-foramen palatine fissure.

**Materials and methods:**

case study with four adult men, all with repaired post-foramen palatine fissure. VPC images through nasal-pharyngoscopy during the emission o f the [a] vowel in a modal and basal recording. The images were studied by four ENTs.

**Results:**

in three subjects there was no change in the type of VPC considering the recordings analyzed; the changes which happened to most of the subjects are associated only to the degree of movement of the structures involved, since in the basal recording the movement of the laryngeal lateral walls was kept, the movement of the posterior pharyngeal wall stabilized, the movement of the palatine veil was mildly reduced, and the Passavant fold was evident.

**Conclusions:**

The type of VPC was kept in the four subjects analyzed, when we compared the modal and basal recordings, there was a modification in the degree of movements in the structures involved, making it clear the Passavant Fold.

## INTRODUCTION

The velopharyngeal sphincter (VPS), which works as a valve closing as a sphincter, corresponds to the area of the lateral and posterior pharyngeal walls, being anteriorly bordered by the soft palate^1^^,^^2^.

One of its functions is to separate the nasal cavity from the oropharynx^3^, which is a very important factor in the production of a balanced speech, preventing nasal air to escape during consonant articulation^4^, ^5^, ^6^.

Physiologically speaking, there is a major variety involving its closure mechanism, which can be classified in four different patterns based on the different movement degrees of the palate and pharyngeal wall: coronal, sagittal, circular and circular with the Passavant ridge^7^.

The speech alterations presented by patients with fissures are mainly the consequence of a malfunction of the velopharyngeal mechanism, since velopharyngeal insufficiency can cause an unbalance on oronasal resonance, leading to a predominance of hypernasal voice^8^, ^9^, ^10^.

When surgical treatment does not result in a VPS capable of maintaining proper resonance during speech^9^^,^^11^, speech and hearing therapy can contribute much to the rehabilitation of these patients with fissures^8^^,^^12^^,^^13^, being carried out with the use of vocal techniques.

Today, the basal sound technique is used in functional and organofunctional dysphonias^14^^,^^15^; nonetheless, according to some authors^8^^,^^12^^,^^16^, ^17^, ^18^, ^19^, ^20^, ^21^, ^22^, ^23^ this technique can help VPS closure and, consequently, reduce nasal resonance which is so much present in voices of patients with fissures.

Thus, the present study aims at analyzing VPS closure in male patients with surgically-repaired post-foramen palatine fissures, during the basal sound technique, and comparing it to the closure during utterance in modal register. Thus, we aim at expanding the knowledge about the relations between the use of a basal register and nasopharyngeal physiology.

## CASE PRESENTATION

This study is based on an investigation of cases, aiming at describing and analyzing the results obtained. Raw data (images) were obtained from the Database of the institution of origin, and such study was previously approved by the Ethics in Research Committee of this institution (protocol number 23081.008439/2007-16).

In order to obtain the raw data from the present study, after being taken from the database, the patients underwent the following procedures.

We consider the ethical concerns stemming from the performance of experiments and studies with human beings, meeting the demands from ordinance 196/96 from the National Committee of Ethics in Research (CONEP), by means of having the individual sign a free and informed consent form, guaranteeing all the patients the right to identity and volunteering.

The sample was made up of a group of four male volunteers from the database, whom met the following inclusion criteria: be advised about the study and having signed the TCLE; bearing a surgically-repaired post-foramen palatine fissure; be male; and not having undergone prior speech and hearing treatment in order to avoid muscle conditioning being used to stimulate velopharyngeal closure. Exclusion criteria were: be an adolescent or an elderly - in order to avoid vocal alteration period stemming from vocal change which in men happens between 13 and 15 years of age^24^, as well as presbyphonia - which happens with aging25; not being able to carry out the basal sound technique, considering that every speech therapy technique must be previously taught, and not all patients are able to do all the techniques proposed; have hearing problems, since the auditory feedback plays an important role in voice production26, in such a way that its lack or distortion may impact vocal self-monitoring^27^ and, consequently, compromise the auditory perception of hypernasality and that of basal sound utterance; and have neurological alterations or other malformations.

The following materials were used: Institutional Authorization Form; TCLE; interview protocol; hearing assessment - which was paid for by each patient by means of a referral to the otorhinolaryngologist; ENT assessment through nasopharyngoscopy, studying the velopharyngeal function which used: VHS tape for register, Semp Toshiba 14″ Lumina Line TV set, Sony (VHS) 4 head VCR - SLV – 66 BR, RL −100 Welch Allyn Rhino-Laryngoscope, Kom Lux Light Source; HL2250 optic fiber, image conversion technology from VCR to CDROM for later analysis by means of the Virtual Dub, from Pinacle PCTV; Power Point software.

At first, we did an interview with the patient in order to investigate demographic data, diagnosis at birth, data regarding surgery and whether or not the patient underwent prior speech therapy, among other aspects, with the aim of selecting patients through inclusion and exclusion criteria.

Later on, the patients received a verbal explanation regarding the importance of the study at hand, the importance of signing the TCLE, inclusion and exclusion criteria of the treatment technique to be used. Here, patients were taught to do the basal sound technique, being then monitored by a speech therapist.

Each patient brought a copy of their auditory hearing, previously requested by the ENT physician, based on the exclusion criteria.

Of the 14 volunteers (five males and nine females) who participated in the study, ten were taken off for not fitting either the inclusion and/or exclusion criteria, leaving four male individuals with 19, 24, 24 and 26 years of age who underwent otorhinolaryngological evaluation by means of a nasopharyngoscopy exam with the study of the velopharyngeal function.

This evaluation happened at two times: in the first, the individuals were instructed to utter the vowel [a] sustained in the modal register, in regular tone and intensity in the maximum phonation time^27^. At a second time, the participants uttered a basal sound during a maximum phonation time^16^^,^^28^. During such evaluation, we recorded the images of the VPS closure area.

The images selected from the nasopharyngoscopy exam were frozen, digitalized and transferred to the Power Point presentation software. Each image was arbitrarily sized into 12.5 × 10cm^2^. This does not imply the fact that the patients' anatomy presented these dimensions, but rather that the images were enlarged in order to enhance visualization. In order to calculate VPS closure areas, we used Vector Works - used in architecture to calculate a given area automatically over the arbitrary dimensions of 12.5 × 10cm^2^.

After the stages described above and which data was stored in the Database, we started the procedures of the present study.

In order to analyze the type of velopharyngeal closure and degree of movement of velopharyngeal structures the patients performed, before and after the basal sound technique, the images of before and during the basal sound utterance were assessed by three otorhinolaryngologists, and for results we considered their common opinion or the prevailing one, among the assessment of the examiners. We used a specific evaluation protocol regarding the type of VPS closure.

The type of velopharyngeal closure was classified taking into account the following closure patterns^7^:

– Coronal: a clearer participation of the soft palate;
– Sagittal: a clearer participation of the lateral pharyngeal walls;
– Circular with Passavant ridge: clear participation of the soft palate and lateral pharyngeal walls, including the posterior pharyngeal wall with the Passavant ridge.

Each ENT physician judged independently from the others (alone), in order to avoid biased results, using the specific protocol and without knowing whether the image assessed was related to before or during basal sound utterance, not even if they corresponded to the same patient. Moreover, each image was presented twice to each examiner, in other words, the images of before and during the basal sound from each patient appeared twice for each ENT, randomly, in such a way that each examiner examined the same image twice, without knowing it. Moreover, the examiners were not aware of the study goals. These factors guaranteed greater result reliability.

A fourth ENT examiner was called in case the first three examiners had the same opinion, thus having each image examined six times.

After the evaluations, because of the reduced number of patients, it was not possible to obtain a statistical treatment of the data and the data found was analyzed in a qualitative way, which characterizes this study as a case study.

The velopharyngeal closure images before and during the utterance in baseline register are exposed in Figure 1, Figure 2, Figure 3, Figure 4, Figure 5, Figure 6, Figure 7, Figure 8.

**Figure 1:**
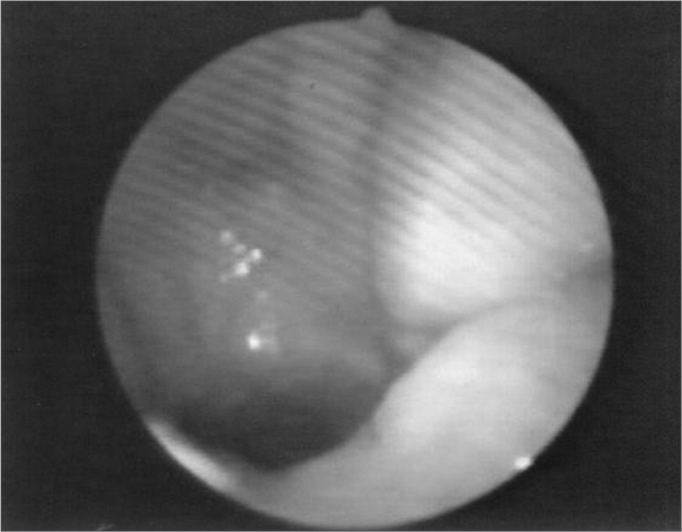
Patient1: VPS closure in modal register

**Figure 2:**
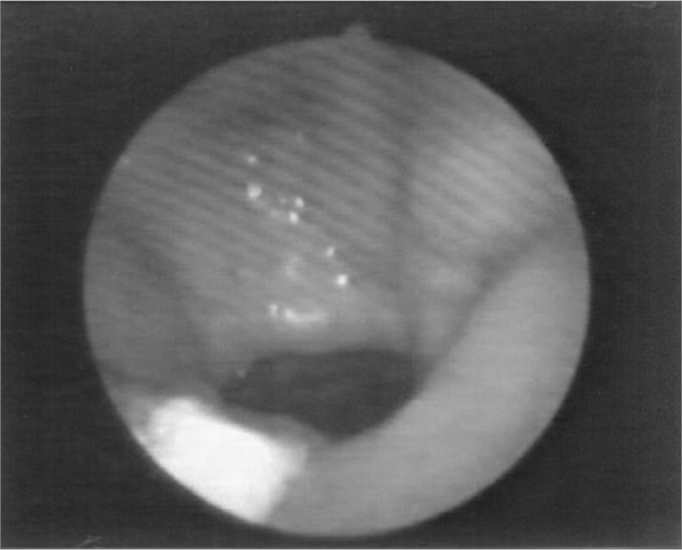
Patient1: VPS closure in basal register

**Figure 3:**
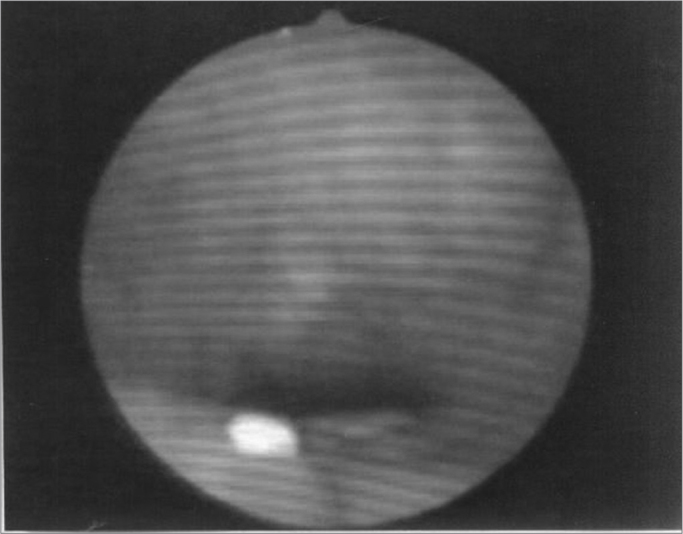
Patient2: VPS closure in modal register

**Figure 4:**
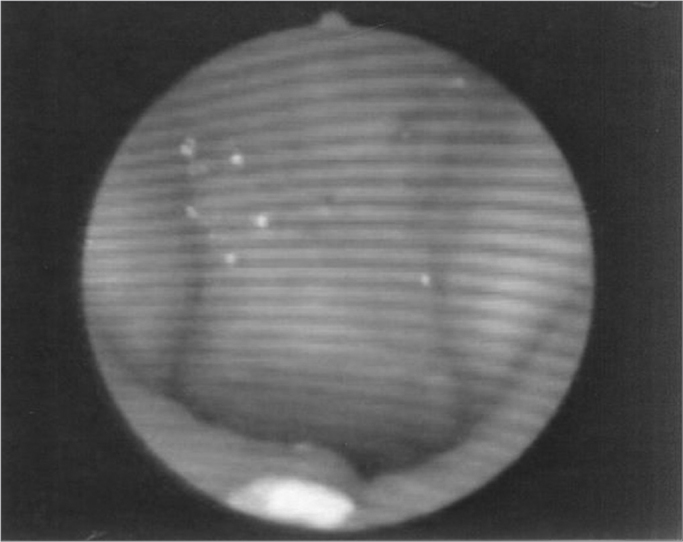
Patient2: VPS closure in basal register

**Figure 5:**
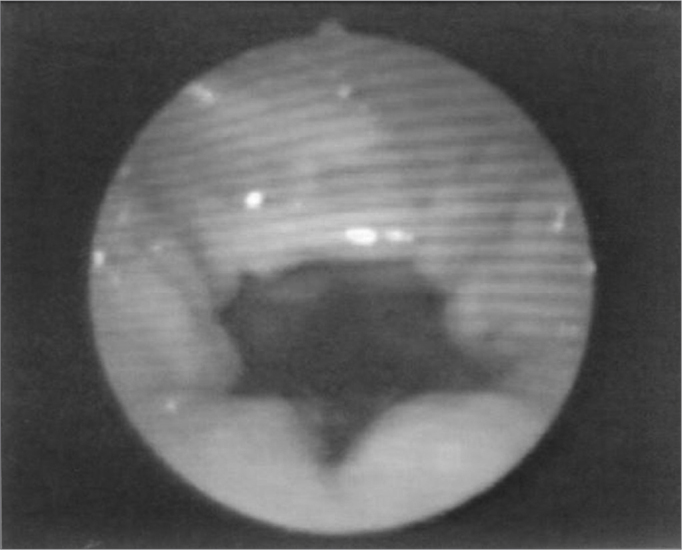
Patient3: VPS closure in modal register

**Figure 6:**
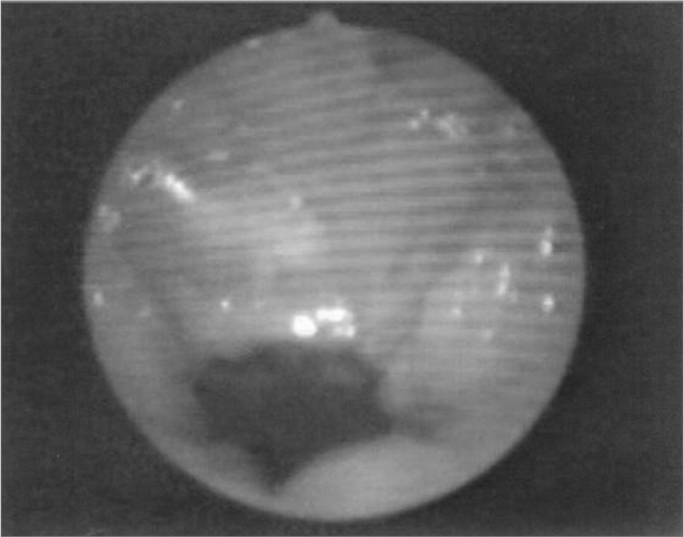
Patient3: VPS closure in basal register

**Figure 7:**
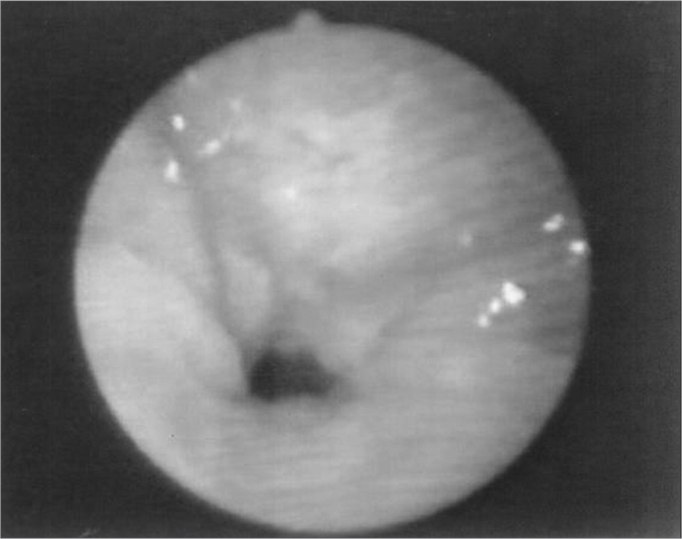
Patient4: VPS closure in modal register

**Figure 8:**
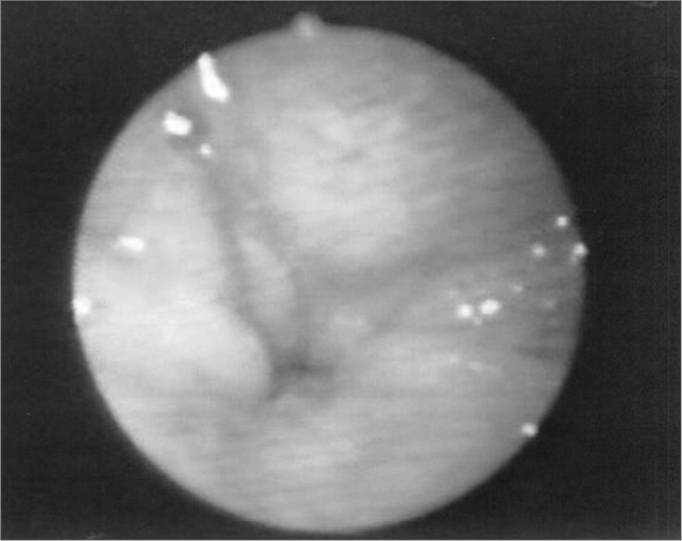
Patient4: VPS closure in basal register

The results from the images examined by the ENT examiners, classified according to the type of velopharyngeal closure done by the patients and the movements of the VPS structures before and during the basal sound utterance are on Table 1, Table 2. The results from the velopharyngeal closure area, calculated by the Vector Works software are exposed on Table 3.

**Table 1:** Results from the VPS image analysis, according to the type of velopharyngeal closure and movement of the structures before and during the basal sound utterance, per patient.

Patient	Type of closure	Lateral pharyngeal wall movement	Posterior pharyngeal all movement	Soft palate movement	Passavant fold presence	
1	Modal	Coronal	Medium	Mild	Medium	Null
	Basal	Sagittal	Medium	Medium	Mild	Medium
2	Modal	Coronal	Medium	Null	Medium	Null
	Basal	Coronal	Null	Null	Medium	Null
3	Modal	Sagittal	Mild	Medium	Mild	Mild
	Basal	Sagittal	Medium	Medium	Mild	Medium
4	Modal	Circular with Passavant fold	Intense	Intense	Medium	Intense
	Basal	Circular with Passavant ridge	Intense	Intense	Medium	Intense

**Table 2:** Preponderance of results from the VPS image analysis, according to the type of velopharyngeal closure and movement of the structures, before and during the basal sound utterance, in the group (summary of the Table 1 results)

Register	Closure type	Lateral pharyngeal wall movement	Posterior pharyngeal wall movement	Soft palate movement	Passavant fold present
Modal	Coronal	Medium	Varied	Medium	Null
Basal	Sagittal	Medium	Medium	Mild-medium	Medium

**Table 3:** Results from the velopharyngeal closure area, calculated by the Vector Works software.

Patient		VPS closure area	VPS closure gain
1	Modal	7,87 cm^2^	2,03 cm^2^
	Basal	5,84 cm^2^	
2	Modal	3,35 cm^2^	1,89 cm^2^
	Basal	1,89 cm^2^	
3	Modal	14,22 cm^2^	1,41 cm^2^
	Basal	12,81 cm^2^	
4	Modal	1,25 cm^2^	1,25 cm^2^
	Basal	0 cm^2^	

## DISCUSSION

Velopharyngeal closure is done by the palate soft tissue tension and by its elevation towards the pharyngeal walls, which also move towards it^3^. Nonetheless, as it happened to those individuals with palatine fissure, the VPS forming muscles were found altered in their anatomy and physiology, not providing for a normal functioning of the muscles and bones^1^^,^^9^^,^^29^^,^^30^, it is considered that the closures studied here were “closure attempt or trend”^31^, in order to understand that the cases studied were from individuals with this deformity and total VPS closure not always occurred in the two registers studied.

According to the literature, the subjects with palatine fissure had very reduced soft palate shifting^1^. Nonetheless, in the present study, during the modal register utterance, two of the four fissured patients had the coronal type of velopharyngeal closure (Table 1), which is more clearly active in the soft palate^7^, and one presented a circular type of Passavant ridge (Table 1), which has a clear participation in the soft palate and lateral pharyngeal walls, including the posterior pharyngeal wall with the Passavant ridge^7^.

Still, fissured subjects had an increase in the circular or sagittal closure type, which leads us to think that a greater participation of the pharyngeal lateral walls could compensate for the velopharyngeal dysfunction compensation^31^. Nonetheless, in the present study, only one patient presented sagittal-type velopharyngeal closure, and the circular type of closure was not found in any patient from the modal register (Table 1).

Notwithstanding, the fact that two fissured patients had the type of coronal closure in modal emission in the present study (Table 1) agrees with the results found in a study in which they analyzed the findings from the nasal air emission and nasopharyngoscopy exams in 21 individuals operated because of incisive post and transforaminal fissures, noticing a predominance of the coronal-type of closure (48%)^31^. As far as the modal register goes, the present study also agrees with another study which found the coronal as the predominant type of velopharyngeal closure, both in the modal and in the basal modal register^16^. As far as the basal register goes, this study found a predominantly sagittal velopharyngeal closure (Table 2), diverging from the previously mentioned study^16^.

In a general way, what we found in this study, comparing velopharyngeal closure with the modal register was that the type of closure was kept the same in most of the patients, and there were changes to the degree of movement of the structures involved.

By analyzing the movement of VPS structures in the group and comparing it to the modal register utterance, we noticed that during the basal register utterance the movement of the posterior pharyngeal wall was greater and more stable, as well as the presence of the Passavant fold which was also greater. The soft palate movement was mildly reduced and the lateral pharyngeal wall movements were maintained.

Based on the findings then, it is possible to suppose that during the basal sound the Passavant ridge and the posterior pharyngeal wall make up for the fact that the patients with palatine fissure have reduced soft palate movement1, increasing VPS closure^8^^,^^12^^,^^16^, ^17^, ^18^, ^19^, ^20^, ^21^, ^22^, ^23^ through these two structures.

We can explain this increase in the movement of the posterior pharyngeal wall and the presence of the Passavant ridge, caused by a change in vocal register, caused by the change in vocal register through the statement of some authors that the vocal production requires the synchrony between the VPS valve closure and that of the vocal fold valves, in other words, there is a very clear physiological inter-relation between the larynx and the soft palate^1^^,^^32^. Since the basal register causes greater glottal closure^33^^,^^34^ it is possible that there has been a greater VPS muscle activity in the patients in this study, activating even further the posterior pharyngeal wall and the Passavant ridge.

Many studies associated with the basal register reinforce this possibility with the study carried out in adult females without laryngeal changes with the goal of checking the degree of constriction of the nasal pars of the larynx during utterance in basal register when compared to the modal register. The results from this study found greater constriction of the pharynx nasal pars during basal recording when compared to the modal register, and also greater movement amplitude of the soft palate and lateral pharyngeal wall and greater contraction of the uvula muscle during basal register^16^.

Other authors mention the use of the basal sound technique in order to have a greater VPS closure, since such technique can act isometrically on the nasal pars of the pharynx^8^^,^^12^, as it happened in the patients of the present study, confirmed by the gain, in this area, in the VPS closure during utterance in basal register (Table 3).

In a study carried out with the goal of checking the movement of the velopharyngeal sphincter structures during basal sound utterance in order to reduce hypernasality, was found during the technique an important medialization of the lateral pharyngeal walls, anteriorization of the posterior pharyngeal wall, as well as soft palate and uvula elevation - causing total closure of the VPS from the assessed patient^20^.

Still, in the paper which aimed at studying the basal sound efficacy on the VPS closure in five adult individuals with surgically-repaired post-foramen fissure and without prior speech treatment, we noticed a mean gain of 4.25 cm^2^ of closure in relation to the VPS closure before the basal sound in four of the five cases evaluated (12×10cm images and measures taken from these image dimensions). Thus having that the basal sound technique is very efficient to stimulate muscle mobility and maintain muscle behavior of the structures which make up the VPS, reducing the air escape area which causes hypernasality in the cases of inadequate velopharyngeal closure^22^, and this was also shown in this study when the VPS closure areas were compared during basal sound with the modal register in all the patients (Table 3).

Through the results obtained in this study^16^, we can state that the increase in nasopharyngeal constriction, involving the VPS, during basal register is due to the fact that, in this register the larynx is lowered, increasing pharyngeal vertical dimensions, and it is necessary to perform motor adjustments in the entire vocal tract in order to maintain proper resonance to the individual's voice emission. This constriction increase in the nasopharynx during vocal emission in basal register was also seen in patients of the present study.

It is also important to consider the study which aimed at studying the metallic voice considering the adjustments which happen in the velopharyngeal, pharynx and larynx during emission. In the present study, the authors found, among the results, ten subjects who had soft palate lowering during the metal emission and, among these ten individuals, nine had laryngeal elevation together with soft palate lowering. Thus, they inferred that such results can be associated to the physiological interconnection between these two adjustments, partially caused by the palatopharyngeal muscle^35^.

The physiological interconnection caused by the palatopharyngeal muscle is one of the factors believed to have influenced the greater movement of the posterior pharyngeal wall and that of the VPS Passavant ridge during basal register, when compared to the modal register of this study, having seen that this muscle acts as palate elevator, it lowers the pharynx and the soft palate^1^^,^^35^ besides being part of the Passavant ridge^1^.

During emission in the basal register of patient 1, we could notice that when compared to the emission in modal register, there was a reduction in soft palate movement (Table 1). Such fact can be explained by the fact that the patient had coronal closure, which has a greater participation in the soft palate^7^, during modal production, presenting the sagittal closure type, it is done with a greater participation of the lateral pharyngeal walls^7^, during basal utterance.

It is also believed that movement changes of the structures which make up the VPS during basal sound production, compared to the utterance in modal register have also occurred vertically, compared to the modal register utterance, also happened vertically, since the VPS acts tridimentionally^1^^,^^2^^,^^10^^,^^29^.

These statements are relevant when one analyzes a study which found in the utterance during basal register from some participating subjects that the contact point between the soft palate and the posterior pharyngeal wall visually happened at a higher point in the cranium-caudal axis, in other words, it moved vertically^16^.

Based on the statement that the soft palate mobilization and the closure created by the continuous utterance in basal sound does not depend on the type of velopharyngeal closure^16^, it is believed that, even in the absence of, changes in the velopharyngeal closure during the utterance of a basal sound, there was a greater movement of some velopharyngeal structures, which can be responsible for the greater VPS closure during this type of utterance, reducing nasal resonance so much present and harmful to the communication of palatine fissure patients^8^^,^^12^^,^^16^, ^17^, ^18^, ^19^, ^20^, ^21^, ^22^, ^23^, which could be confirmed by the gain in the area of VPS closure seen during basal register, when compared to modal, in all analyzed patients (Table 3).

Still, we must mention that, when the images from each patient were placed side by side, one can see a great movement of the structures involved in VPS closure during basal register, in all the patients, greater than the result of the movement examined by the physicians. This fact is likely due to a blind exam, image per image, without the possibility of comparing patients, done by the otorhinolaryngologists in the present study.

## CONCLUSIONS

Based on this case study, we can conclude that the type of velopharyngeal sphincter closure was maintained in three of the four patients analyzed, when comparing modal to basal register.

The modifications which happen in most of the cases studied is associated with the degree of movement of the structures involved, since in basal register the movement of the lateral pharyngeal walls was maintained, the movement of the posterior pharyngeal wall was stabilized, soft palate movement reduced a little and the Passavant ridge was made more clear.
